# The genome sequence of the Orange Ripple Bryozoan, *Schizoporella japonica* Ortmann, 1890 (Cheilostomatida: Schizoporellidae)

**DOI:** 10.12688/wellcomeopenres.27394.1

**Published:** 2026-08-27

**Authors:** John Bishop, Marine Biological Association Genome Acquisition Lab, Darwin Tree of Life Barcoding Collective, Wellcome Sanger Institute Tree of Life Management, Samples and Laboratory team, Wellcome Sanger Institute Scientific Operations: Sequencing Operations, Wellcome Sanger Institute Tree of Life Core Informatics team, Tree of Life Core Informatics collective, Darwin Tree of Life Consortium

**Affiliations:** 1 The Marine Biological Association, Plymouth, England, UK

**Keywords:** Schizoporella japonica, Orange Ripple Bryozoan, genome sequence, chromosomal, Cheilostomatida

## Abstract

We present a genome assembly from a specimen of *Schizoporella japonica* (Orange Ripple Bryozoan; Bryozoa; Gymnolaemata; Cheilostomatida; Schizoporellidae). The genome sequence has a total length of 255.53 megabases. Most of the assembly (99.03%) is scaffolded into 19 chromosomal pseudomolecules. The mitochondrial genome has also been assembled, with a length of 18.45 kilobases. This assembly was generated as part of the Darwin Tree of Life project, which produces genomes for eukaryotic species found in Britain and Ireland.

## Species taxonomy

Eukaryota; Opisthokonta; Metazoa; Eumetazoa; Bilateria; Protostomia; Spiralia; Lophotrochozoa; Bryozoa; Gymnolaemata; Cheilostomatida; Flustrina; Schizoporelloidea; Schizoporellidae; *Schizoporella*; *Schizoporella japonica* Ortmann, 1890 (NCBI:txid2589381)

## Background

*Schizoporella japonica* Ortmann, 1890 is an encrusting cheilostome bryozoan in the family Schizoporellidae. Colonies are pale pink to vivid orange-red and form calcified sheets, often with raised edges or foliose lobes. The zooids are usually about twice as long as broad and have a densely perforated frontal wall. The species is hermaphroditic, and embryos are brooded in external ovicells. Its ciliated, non-feeding larvae usually settle and metamorphose within hours of release ( Loxton *et al.*, 2017; Ryland *et al.*, 2014).

The species is native to the north-west Pacific, from China to Japan. In its native range, it occurs mainly on intertidal rocks, boulders and shellfish, but introduced populations commonly colonise artificial surfaces, including pontoons, boat hulls, aquaculture equipment and marine-energy devices. It was introduced to the Pacific coast of North America, probably with Pacific oysters, and was present in Britain by 2009. In Great Britain and Ireland, it has a widely discontinuous distribution, with many records from Scottish marinas and harbours. Vessel fouling and the movement of aquaculture stock and equipment are likely to assist its spread. Because it can dominate fouling communities, overgrow native organisms and affect marine industries, it is regarded as invasive in Great Britain and Ireland ( Loxton *et al.*, 2017; Ryland *et al.*, 2014).

We present a chromosome-level genome sequence for *Schizoporella japonica.* The assembly was produced using the Tree of Life pipeline using a specimen collected from Mill Bay, Plymouth, Devon, UK ( Figure 1)

**Figure 1.**
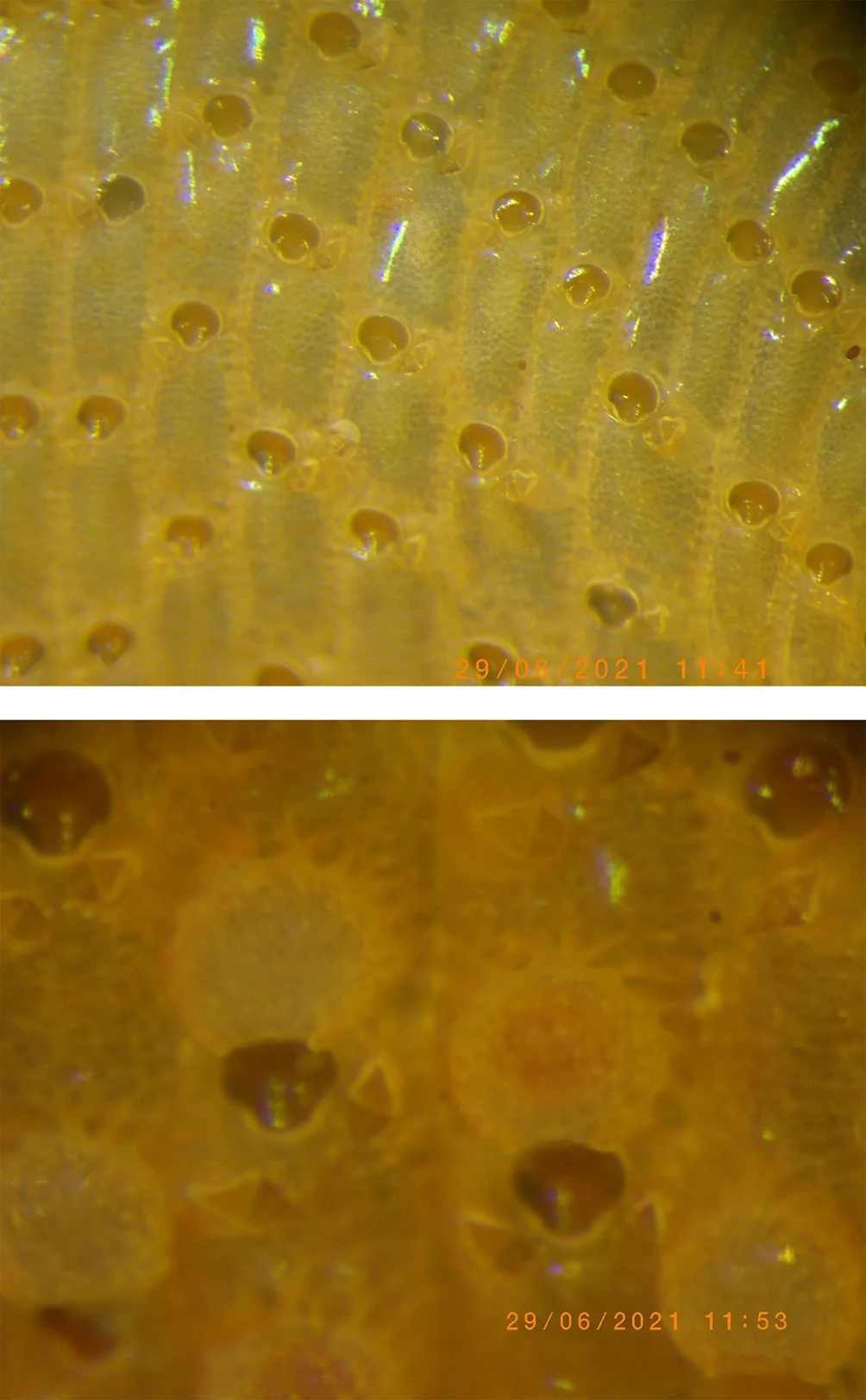
The *Schizoporella japonica* specimen (tzSchJapo1) used for genome sequencing.

## Methods

### Sample acquisition and DNA barcoding

The specimen used for genome sequencing was a *Schizoporella japonica* (specimen ID MBA-210623-002A, ToLID tzSchJapo1; Figure 1). The same specimen was also used for Hi-C sequencing and RNA sequencing. It was collected from Mill Bay, Plymouth, Devon, UK (latitude 50.3669, longitude −4.1552) on 2021-06-23 by John Bishop and Christine Wood and identified by John Bishop.

The initial identification was verified by an additional DNA barcoding process according to the framework developed by Twyford *et al.* (2024). A small sample was dissected from the specimen and stored in ethanol, while the remaining parts were shipped on dry ice to the Wellcome Sanger Institute (WSI) (see the protocol). The tissue was lysed, the COI marker region was amplified by PCR, and amplicons were sequenced and compared to the BOLD database, confirming the species identification. Following whole genome sequence generation, the relevant DNA barcode region was also used alongside the initial barcoding data for sample tracking at the WSI ( Twyford *et al.*, 2024). The standard operating procedures for Darwin Tree of Life barcoding are available on protocols.io.

### Nucleic acid extraction

Detailed protocols for nucleic acid extraction developed at the Wellcome Sanger Institute (WSI) Tree of Life Core Laboratory are available on protocols.io ( Howard *et al.*, 2025). The tzSchJapo1 specimen was weighed and triaged to determine the appropriate extraction protocol. For high molecular weight (HMW) DNA extraction, 28.0 mg of tissue was homogenised by powermashing using a PowerMasher II tissue disruptor. The Automated MagAttract v.1 extraction protocol was used, followed by fragmentation. We used centrifuge-mediated fragmentation to produce DNA fragments in the 8–10 kb range, following the Covaris g-TUBE protocol for ultra-low input (ULI). Sheared DNA was purified by manual SPRI (solid-phase reversible immobilisation). The concentration of the sheared and purified DNA was assessed using a Nanodrop spectrophotometer and Qubit Fluorometer using the Qubit dsDNA High Sensitivity Assay kit. Fragment size distribution was evaluated by running the sample on the FemtoPulse system. For this sample, the final post-shearing DNA had a Qubit concentration of 2.1 ng/μL, with a fragment size of 8.8 kb. The 260/280 spectrophotometric ratio was 1.26, and the 260/230 ratio was 0.78.

RNA was extracted from modular colony tissue of the tzSchJapo1 specimen in the Tree of Life Laboratory at the WSI using the RNA Extraction: Automated MagMax™ *mir*Vana protocol. The RNA concentration was assessed using a Nanodrop spectrophotometer and a Qubit Fluorometer using the Qubit RNA Broad-Range Assay kit. Analysis of the integrity of the RNA was done using the Agilent RNA 6000 Pico Kit and Eukaryotic Total RNA assay.

### PacBio HiFi library preparation and sequencing

Library preparation and sequencing were performed at the WSI Scientific Operations core. Prior to library preparation, the DNA was fragmented to ~10 kb. Ultra-low-input (ULI) libraries were prepared using the PacBio SMRTbell® Express Template Prep Kit 2.0 and gDNA Sample Amplification Kit. Samples were normalised to 20 ng DNA. Single-strand overhang removal, DNA damage repair, and end-repair/A-tailing were performed according to the manufacturer’s instructions, followed by adapter ligation. A 0.85× pre-PCR clean-up was carried out with Promega ProNex beads.

The DNA was evenly divided into two aliquots for dual PCR (reactions A and B), both following the manufacturer’s protocol. A 0.85× post-PCR clean-up was performed with ProNex beads. DNA concentration was measured using a Qubit Fluorometer v4.0 (Thermo Fisher Scientific) with the Qubit HS Assay Kit, and fragment size was assessed on an Agilent Femto Pulse Automated Pulsed Field CE Instrument (Agilent Technologies) using the gDNA 55 kb BAC analysis kit. PCR reactions A and B were then pooled, ensuring a total mass of ≥500 ng in 47.4 μl.

The pooled sample underwent another round of DNA damage repair, end-repair/A-tailing, and hairpin adapter ligation. A 1× clean-up was performed with ProNex beads, followed by DNA quantification using the Qubit and fragment size analysis using the Agilent Femto Pulse. Size selection was performed on the Sage Sciences PippinHT system, with target fragment size determined by Femto Pulse analysis (typically 4–9 kb). Size-selected libraries were cleaned with 1.0× ProNex beads and normalised to 2 nM before sequencing.

The sample was sequenced using the Sequel IIe system (Pacific Biosciences, California, USA). The concentration of the library loaded onto the Sequel IIe was in the range 40–135 pM. SMRT Link software, a PacBio web-based workflow manager, was used to set up and monitor the run, and to perform primary and secondary analysis of the data upon completion.

### Hi-C

***Sample preparation and crosslinking***

The Hi-C sample was prepared from 20–50 mg of frozen tissue from the tzSchJapo1 sample using the Arima-HiC v2 kit (Arima Genomics). Following the manufacturer’s instructions, tissue was fixed and DNA crosslinked using TC buffer to a final formaldehyde concentration of 2%. The tissue was homogenised using the Diagnocine Power Masher-II. Crosslinked DNA was digested with a restriction enzyme master mix, biotinylated, and ligated. Clean-up was performed with SPRISelect beads before library preparation. DNA concentration was measured with the Qubit Fluorometer (Thermo Fisher Scientific) and Qubit HS Assay Kit. The biotinylation percentage was estimated using the Arima-HiC v2 QC beads.

***Hi-C library preparation and sequencing***

Biotinylated DNA constructs were fragmented using a Covaris E220 sonicator and size selected to 400–600 bp using SPRISelect beads. DNA was enriched with Arima-HiC v2 kit Enrichment beads. End repair, A-tailing, and adapter ligation were carried out with the NEBNext Ultra II DNA Library Prep Kit (New England Biolabs), following a modified protocol where library preparation occurs while DNA remains bound to the Enrichment beads. Library amplification was performed using KAPA HiFi HotStart mix and a custom Unique Dual Index (UDI) barcode set (Integrated DNA Technologies). Depending on sample concentration and biotinylation percentage determined at the crosslinking stage, libraries were amplified with 10–16 PCR cycles. Post-PCR clean-up was performed with SPRISelect beads. Libraries were quantified using the AccuClear Ultra High Sensitivity dsDNA Standards Assay Kit (Biotium) and a FLUOstar Omega plate reader (BMG Labtech).

Prior to sequencing, libraries were normalised to 10 ng/μL. Normalised libraries were quantified again to create equimolar and/or weighted 2.8 nM pools. Pool concentrations were checked using the Agilent 4200 TapeStation (Agilent) with High Sensitivity D500 reagents before sequencing. Sequencing was performed using paired-end 150 bp reads on the Illumina NovaSeq 6000.

### RNA library preparation and sequencing

Libraries were prepared using the NEBNext® Ultra II Directional RNA Library Prep Kit for Illumina (New England Biolabs), following the manufacturer’s instructions. Poly(A) mRNA was isolated from 100 ng of total RNA using oligo (dT) beads, converted to cDNA, and uniquely indexed; 14 PCR cycles were performed. Libraries were prepared with an intended fragment size of 100–300 bp. Libraries were quantified, normalised, and pooled equimolarly to a final concentration of 2.8 nM before dilution to 150 pM for loading. Sequencing was carried out on the Illumina NovaSeq 6000, generating paired-end reads.

### Genome assembly

Prior to assembly of the PacBio HiFi reads, a database of *k*-mer counts ( *k* = 31) was generated from the filtered reads using FastK. GenomeScope2 ( Ranallo-Benavidez *et al.*, 2020) was used to analyse the *k*-mer frequency distributions, providing estimates of genome size, heterozygosity, and repeat content.

The HiFi reads were assembled using Hifiasm ( Cheng *et al.*, 2021) with the --primary option. The Hi-C reads ( Rao *et al.*, 2014) were mapped to the primary contigs using bwa-mem2 ( Vasimuddin *et al.*, 2019), and the contigs were scaffolded in YaHS ( Zhou *et al.*, 2023) with the --break option for handling potential misassemblies. The scaffolded assemblies were evaluated using Gfastats ( Formenti *et al.*, 2022), BUSCO ( Manni *et al.*, 2021) and MerquryFK ( Rhie *et al.*, 2020).

The mitochondrial genome was assembled using OATK ( Zhou *et al.*, 2025). The MitoHiFi reference was the mitochondrial genome of *Celleporella hyalina* (NC_018344.1).

### Assembly curation

The assembly was decontaminated using the Assembly Screen for Cobionts and Contaminants ( ASCC) pipeline. TreeVal was used to generate the flat files and maps for use in curation. Manual curation was conducted primarily in PretextView and HiGlass ( Kerpedjiev *et al.*, 2018). Scaffolds were visually inspected and corrected as described by Howe *et al*. (2021). Manual corrections included 20 breaks, 49 joins, and removal of 29 haplotypic duplications. This reduced the scaffold count by 36.1%, increased the scaffold N50 by 57.0%, and reduced the total assembly length by 3.5%. The curation process is described at sanger-tol/curation-resources . PretextSnapshot was used to generate a Hi-C contact map of the final assembly.

### Assembly quality assessment

The MerquryFK tool ( Rhie *et al.*, 2020) was run in a Singularity container ( Kurtzer *et al.*, 2017) to evaluate *k*-mer completeness and assembly quality for the primary and alternate haplotypes using the *k*-mer database ( *k* = 31) computed prior to genome assembly. The analysis outputs included assembly QV scores and completeness statistics.

The genome was analysed using the BlobToolKit pipeline, a Nextflow implementation of the earlier Snakemake version ( Challis *et al.*, 2020). PacBio reads were aligned to the assembly using minimap2 ( Li, 2018) and processed with SAMtools ( Danecek *et al.*, 2021) to generate coverage tracks. The pipeline runs BUSCO ( Manni *et al.*, 2021) using lineages identified from the NCBI Taxonomy ( Schoch *et al.*, 2020). For the three basal BUSCO lineages, eukaryota, bacteria and archaea, BUSCO genes are aligned to the UniProt Reference Proteomes database ( Bateman *et al.*, 2023) using DIAMOND BLASTp ( Buchfink *et al.*, 2021). The genome assembly is divided into chunks based on the density of BUSCO genes from the closest taxonomic lineage, and each chunk is aligned to the UniProt Reference Proteomes database using DIAMOND BLASTx. Sequences without DIAMOND BLASTx hits are extracted using seqtk and aligned to the NT database using BLASTn ( Altschul *et al.*, 1990). The outputs are consolidated into a BlobDir for visualisation in BlobToolKit. The BlobToolKit pipeline was developed using nf-core tooling ( Ewels *et al.*, 2020) and MultiQC ( Ewels *et al.*, 2016), with containerisation through Docker ( Merkel, 2014) and Singularity ( Kurtzer *et al.*, 2017).

## Genome sequence report

### Sequence data

PacBio sequencing of the *Schizoporella japonica* specimen generated 21.08 Gb (gigabases) from 2.47 million reads, which were used to assemble the genome. GenomeScope2.0 analysis using a *p* = 2 model gave a *k*-mer-based genome size estimate of 221.44 Mb, with a heterozygosity of 2.60% and repeat content of 27.77% ( Figure 2). These estimates guided expectations for the assembly. Based on the estimated genome size, the sequencing data provided approximately 84× coverage. Hi-C sequencing produced 102.11 Gb from 338.12 million reads, which were used to scaffold the assembly. RNA sequencing data were also generated and are available in public sequence repositories. Table 1 summarises the specimen and sequencing details.

**Figure 2.**
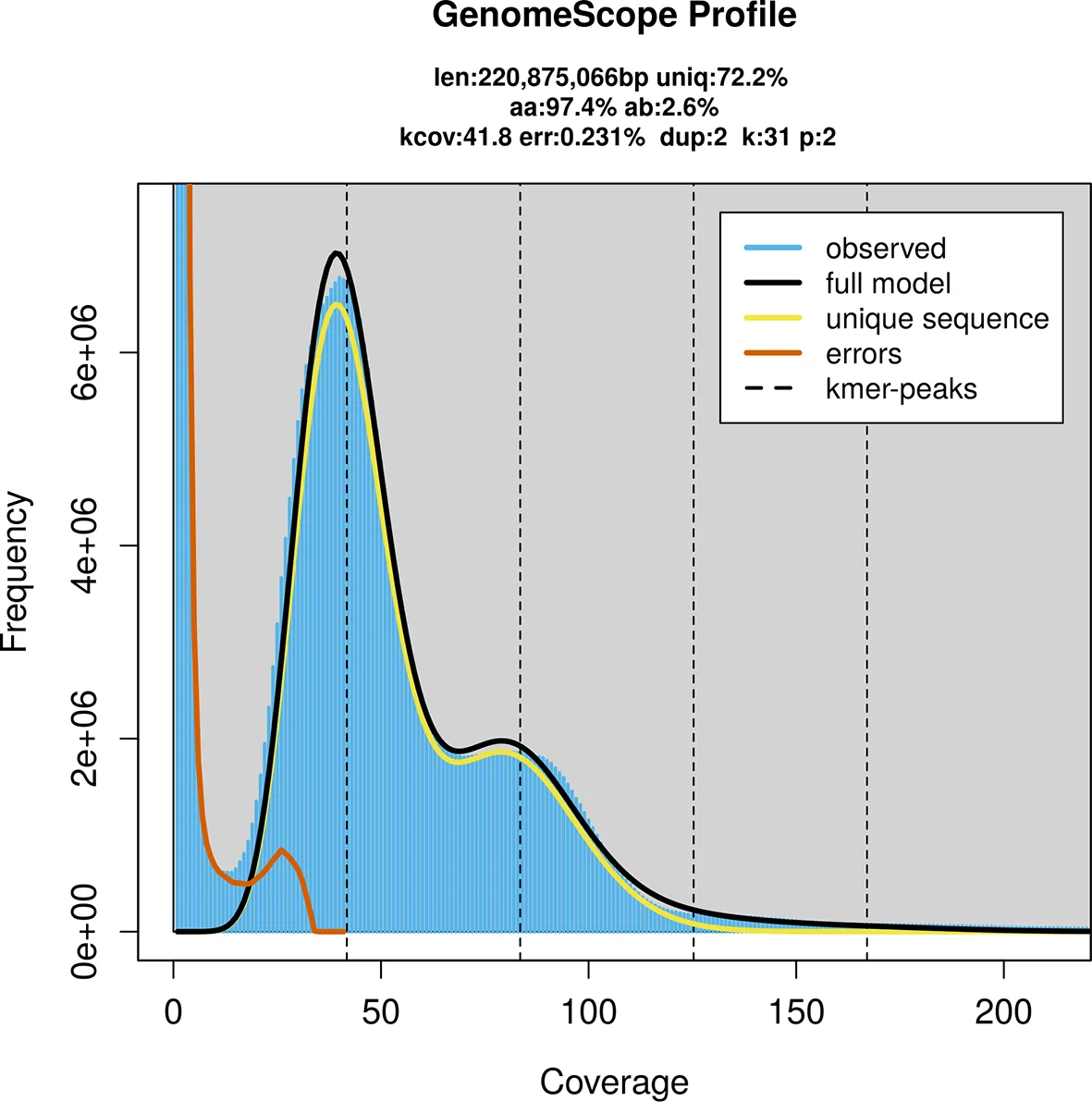
Frequency distribution of *k*-mers generated using GenomeScope2. The plot shows observed and modelled *k*-mer spectra, providing estimates of genome size, heterozygosity, and repeat content based on unassembled sequencing reads.

**Table 1.** Specimen and sequencing data for *Schizoporella japonica* (BioProject PRJEB68091).

Platform	PacBio HiFi	Hi-C	RNA-seq
**ToLID**	tzSchJapo1	tzSchJapo1	tzSchJapo1
**Specimen ID**	MBA-210623-002A	MBA-210623-002A	MBA-210623-002A
**BioSample (source individual)**	SAMEA12231776	SAMEA12231776	SAMEA12231776
**BioSample (tissue)**	SAMEA12231844	SAMEA12231846	SAMEA12231849
**Tissue**	modular colony	modular colony	modular colony
**Instrument**	Sequel IIe	Illumina NovaSeq 6000	Illumina NovaSeq 6000
**Run accessions**	ERR12205308	ERR12245644	ERR12245645
**Read count total**	2.47 million reads	338.12 million read pairs	28.94 million read pairs
**Base count total**	21.08 Gb	102.11 Gb	8.74 Gb

### Assembly statistics

The primary haplotype was assembled, and contigs corresponding to an alternate haplotype were also deposited in INSDC databases. The final assembly has a total length of 255.53 Mb in 135 scaffolds, with 198 gaps, and a scaffold N50 of 13.3 Mb ( Table 2).

**Table 2.** Genome assembly data for *Schizoporella japonica.*

Genome assembly	Primary assembly
**Assembly name**	tzSchJapo1.1
**Assembly accession**	GCA_965278275.1
**Alternate haplotype accession**	GCA_965278305.1
**Assembly level**	chromosome
**Span (Mb)**	255.53
**Number of chromosomes**	19
**Number of contigs**	333
**Contig N50**	2.32 Mb
**Number of scaffolds**	135
**Scaffold N50**	13.3 Mb
**Organelles**	Mitochondrial genome: 18.45 kb

Most of the assembly sequence (99.03%) was assigned to 19 chromosomal-level scaffolds. These chromosome-level scaffolds, confirmed by Hi-C data, are named according to size ( Figure 3; Table 3).

**Figure 3.**
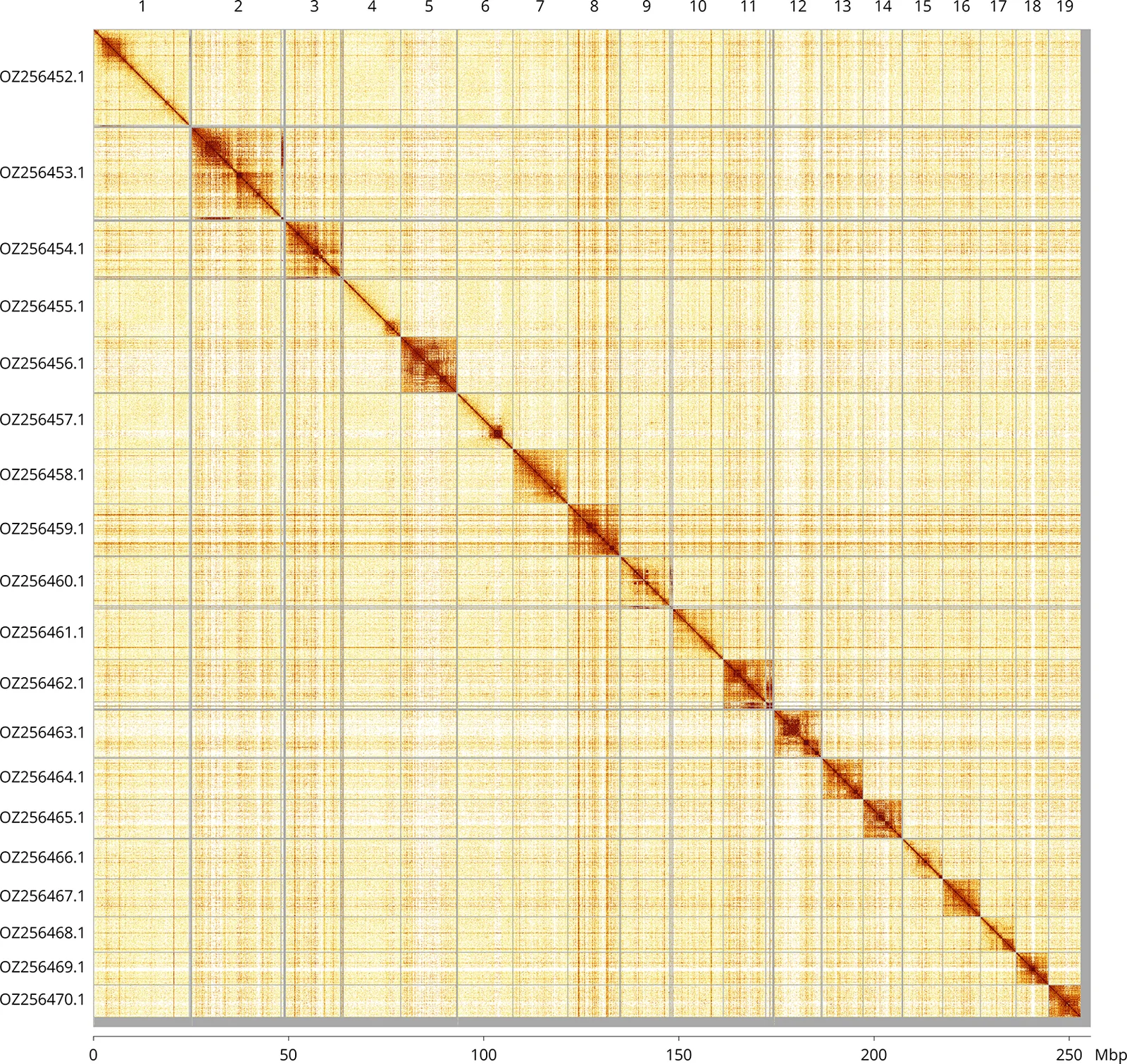
Hi-C contact map of the *Schizoporella japonica* genome assembly. Assembled chromosomes are shown in order of size and labelled along the axes, with a megabase scale shown below. The plot was generated using PretextSnapshot.

**Table 3.** Chromosomal pseudomolecules in the primary genome assembly of *Schizoporella japonica* tzSchJapo1.

INSDC accession	Molecule	Length (Mb)	GC%
OZ256452.1	1	25.16	40.50
OZ256453.1	2	24.06	40.50
OZ256454.1	3	14.89	40
OZ256455.1	4	14.65	41
OZ256456.1	5	14.55	39.50
OZ256457.1	6	14.17	41
OZ256458.1	7	14.07	40
OZ256459.1	8	13.56	39
OZ256460.1	9	13.30	40.50
OZ256461.1	10	13	40.50
OZ256462.1	11	12.97	40
OZ256463.1	12	12.34	40
OZ256464.1	13	10.51	39.50
OZ256465.1	14	10.19	39
OZ256466.1	15	10.18	40.50
OZ256467.1	16	9.70	40
OZ256468.1	17	9.10	40.50
OZ256469.1	18	8.41	40
OZ256470.1	19	8.26	39

The mitochondrial genome was also assembled (length 18.45 kb, OZ256471.1). This sequence is included as a contig in the multifasta file of the genome submission and as a standalone record.

### Assembly quality metrics

The combined primary and alternate assemblies achieve an estimated QV of 63.3. The *k*-mer completeness is 68.12% for the primary assembly, 64.30% for the alternate haplotype, and 99.51% for the combined assemblies ( Figure 4).

**Figure 4.**
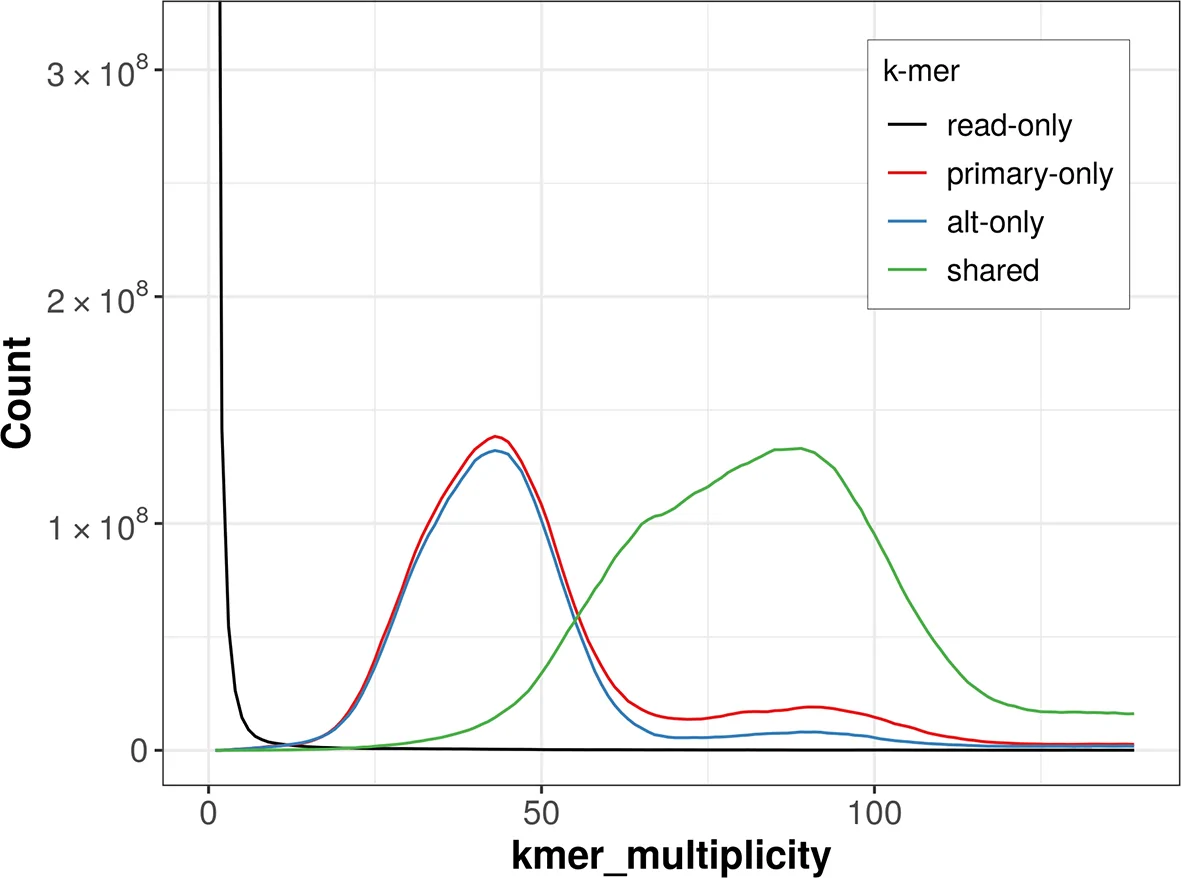
Evaluation of *k*-mer completeness using MerquryFK. This plot illustrates the recovery of *k*-mers from the original read data in the final assemblies. The horizontal axis represents *k*-mer multiplicity, and the vertical axis shows the number of *k*-mers. The black curve represents *k*-mers that appear in the reads but are not assembled. The green curve corresponds to *k*-mers shared by both haplotypes, and the red and blue curves show *k*-mers found only in one of the haplotypes.

BUSCO v.5.7.1 analysis using the metazoa_odb10 reference set ( *n* = 954) identified 85.1% of the expected gene set (single = 83.6%, duplicated = 1.5%). The snail plot in Figure 5 summarises the scaffold length distribution and other assembly statistics for the primary assembly. The blob plot in Figure 6 shows the distribution of scaffolds by GC proportion and coverage.

**Figure 5.**
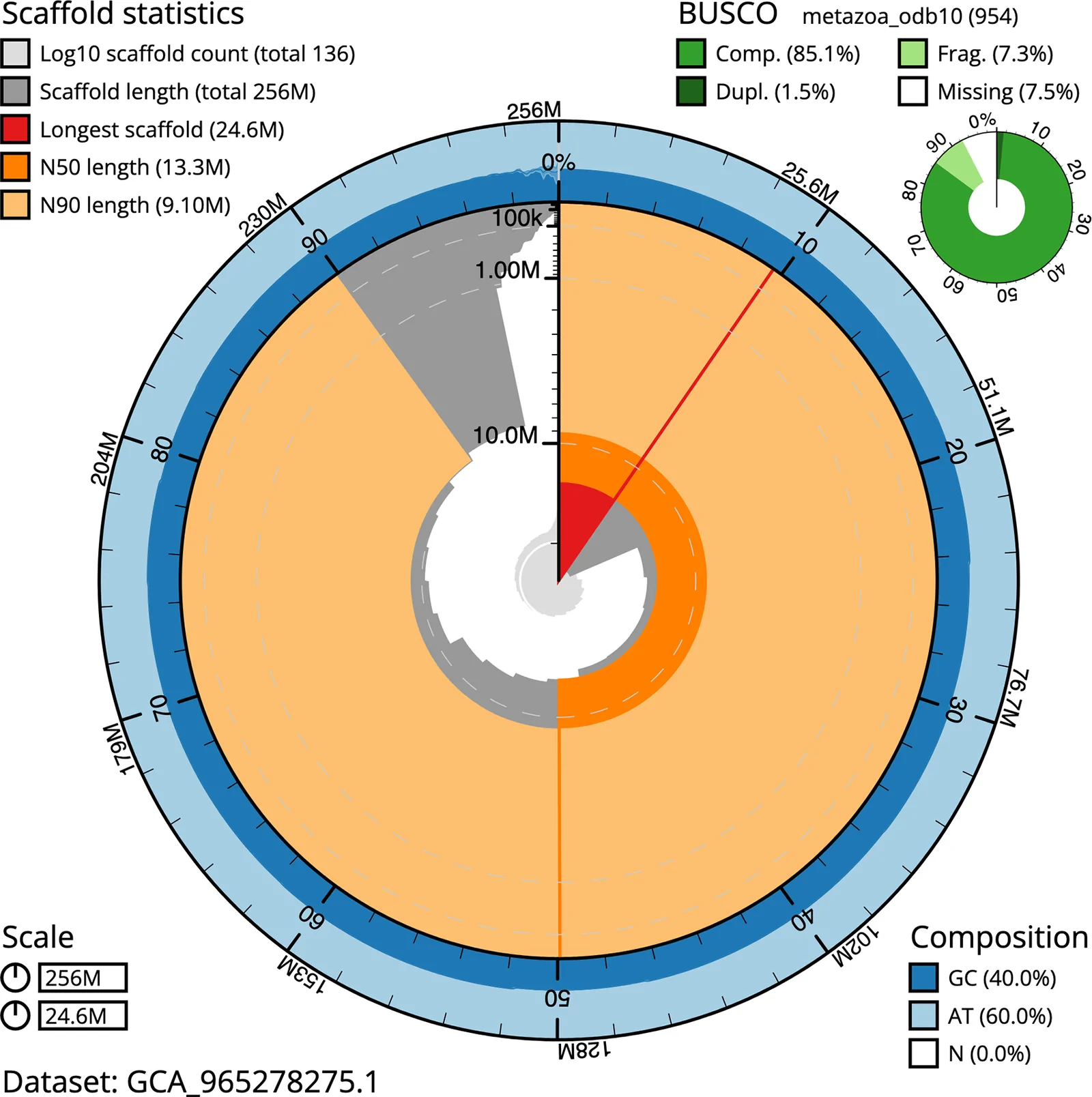
Assembly metrics for tzSchJapo1.1. The BlobToolKit snail plot provides an overview of assembly metrics and BUSCO gene completeness. The circumference represents the length of the whole genome sequence, and the main plot is divided into 1 000 bins around the circumference. The outermost blue tracks display the distribution of GC, AT, and N percentages across the bins. Scaffolds are arranged clockwise from longest to shortest and are depicted in dark grey. The longest scaffold is indicated by the red arc, and the deeper orange and pale orange arcs represent the N50 and N90 lengths. A light grey spiral at the centre shows the cumulative scaffold count on a logarithmic scale. A summary of complete, fragmented, duplicated, and missing BUSCO genes in the metazoa_odb10 set is presented at the top right. An interactive version of this figure can be accessed on the BlobToolKit viewer.

**Figure 6.**
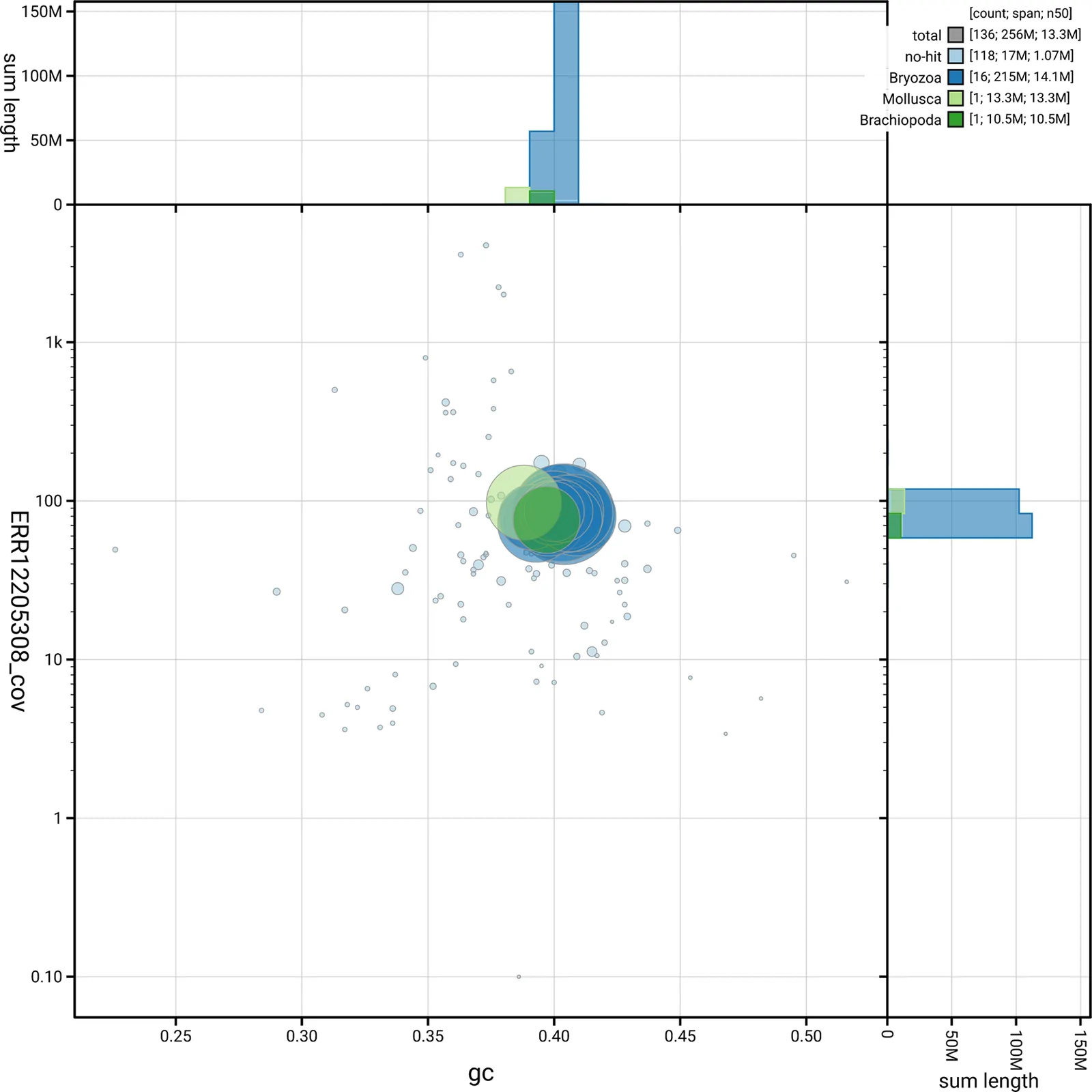
BlobToolKit blob plot for tzSchJapo1.1. The plot shows base coverage (vertical axis) and GC content (horizontal axis). The circles represent scaffolds, with the size proportional to scaffold length and the colour representing phylum membership. The histograms along the axes display the total length of sequences distributed across different levels of coverage and GC content. An interactive version of this figure is available on the BlobToolKit viewer.

Table 4 lists the assembly metric benchmarks adapted from Rhie *et al.* (2021) and the Earth BioGenome Project (EBP) Report on Assembly Standards. The EBP metric, calculated for primary assembly, is **6.C.Q62**, meeting the recommended 6.C.Q40 reference standard.

**Table 4.** Earth Biogenome Project summary metrics for the *Schizoporella japonica* assembly.

Measure	Value	Benchmark
EBP summary (primary)	6.C.Q62	6.C.Q40
Contig N50 length	2.32 Mb	≥ 1 Mb
Scaffold N50 length	13.30 Mb	= chromosome N50
Consensus quality (QV)	Primary: 62.4; alternate: 64.5; combined: 63.3	≥ 40
*k*-mer completeness	Primary: 68.12%; alternate: 64.30%; combined: 99.51%	≥ 90%
BUSCO	C:85.1% [S:83.6%, D:1.5%], F:7.3%, M:7.5%, n:954	S > 90%; D < 5%
Percentage of assembly assigned to chromosomes	99.03%	≥ 90%

**Notes:** The EBP summary uses log10(Contig N50); chromosome-level (C) or log10(Scaffold N50); Q (Merqury QV). BUSCO: C=complete; S=single-copy; D=duplicated; F=fragmented; M=missing; n=orthologues.

**Table 5.** Software versions and sources used for *Schizoporella japonica.*

Software	Version	Source
BLAST	2.14.0	https://ftp.ncbi.nlm.nih.gov/blast/executables/blast+/
BlobToolKit	4.4.5	https://github.com/blobtoolkit/blobtoolkit
BUSCO	5.7.1; 5.4.3	https://gitlab.com/ezlab/busco
bwa-mem2	2.2.1	https://github.com/bwa-mem2/bwa-mem2
DIAMOND	2.1.8	https://github.com/bbuchfink/diamond
fasta_windows	0.2.4	https://github.com/tolkit/fasta_windows
FastK	1.1	https://github.com/thegenemyers/FASTK
GenomeScope2.0	2.0.1	https://github.com/tbenavi1/genomescope2.0
Gfastats	1.3.6	https://github.com/vgl-hub/gfastats
Hifiasm	0.16.1-r375	https://github.com/chhylp123/hifiasm
HiGlass	1.13.4	https://github.com/higlass/higlass
MerquryFK	1.1.0-c1	https://github.com/thegenemyers/MERQURY.FK
Minimap2	2.24-r1122; 2.28-r1209	https://github.com/lh3/minimap2
Oatk	1.0	https://github.com/c-zhou/oatk
MultiQC	1.14; 1.17 and 1.18	https://github.com/MultiQC/MultiQC
Nextflow	23.04.1; 24.10.4	https://github.com/nextflow-io/nextflow
PretextSnapshot	0.0.6	https://github.com/sanger-tol/PretextSnapshot
PretextView	1.0.3	https://github.com/sanger-tol/PretextView
samtools	1.16.1; 1.17; 1.2; 1.21	https://github.com/samtools/samtools
sanger-tol/ascc	0.1.0	https://github.com/sanger-tol/ascc
sanger-tol/blobtoolkit	v0.7.1	https://github.com/sanger-tol/blobtoolkit
sanger-tol/curationpretext	1.4.2	https://github.com/sanger-tol/curationpretext
Seqtk	1.4-r122	https://github.com/lh3/seqtk
Singularity	3.9.0	https://github.com/sylabs/singularity
TreeVal	1.0.0	https://github.com/sanger-tol/treeval
YaHS	1.1a.2	https://github.com/c-zhou/yahs

## Author information

Contributors are listed at the following links:

• Members of the Marine Biological Association Genome Acquisition Lab
• Members of the Darwin Tree of Life Barcoding collective
• Members of the Wellcome Sanger Institute Tree of Life Management, Samples and Laboratory team
• Members of Wellcome Sanger Institute Scientific Operations – Sequencing Operations
• Members of the Wellcome Sanger Institute Tree of Life Core Informatics team
• Members of the Tree of Life Core Informatics collective
• Members of the Darwin Tree of Life Consortium

## Wellcome Sanger Institute – Legal and Governance

The materials that have contributed to this genome note have been supplied by a Darwin Tree of Life Partner. The submission of materials by a Darwin Tree of Life Partner is subject to the **‘Darwin Tree of Life Project Sampling Code of Practice’**, which can be found in full on the Darwin Tree of Life website. By agreeing with and signing up to the Sampling Code of Practice, the Darwin Tree of Life Partner agrees they will meet the legal and ethical requirements and standards set out within this document in respect of all samples acquired for, and supplied to, the Darwin Tree of Life Project. Further, the Wellcome Sanger Institute employs a process whereby due diligence is carried out proportionate to the nature of the materials themselves, and the circumstances under which they have been/are to be collected and provided for use. The purpose of this is to address and mitigate any potential legal and/or ethical implications of receipt and use of the materials as part of the research project, and to ensure that in doing so we align with best practice wherever possible. The overarching areas of consideration are:

• Ethical review of provenance and sourcing of the material
• Legality of collection, transfer and use (national and international)

Each transfer of samples is further undertaken according to a Research Collaboration Agreement or Material Transfer Agreement entered into by the Darwin Tree of Life Partner, Genome Research Limited (operating as the Wellcome Sanger Institute), and in some circumstances, other Darwin Tree of Life collaborators.

## Data Availability

European Nucleotide Archive: Schizoporella japonica ((a bryozoan)). Accession number PRJEB68091; https://identifiers.org/ena.embl/PRJEB68091. The genome sequence is released openly for reuse. The *Schizoporella japonica* genome sequencing initiative is part of the Darwin Tree of Life Project (PRJEB40665) and the Sanger Institute Tree of Life Programme (PRJEB43745). All raw sequence data and the assembly have been deposited in INSDC databases. The genome will be annotated using available RNA-Seq data and presented through the Ensembl pipeline at the European Bioinformatics Institute. Raw data and assembly accession identifiers are reported in Tables 1 and 2. Production code used in genome assembly at the WSI Tree of Life is available at https://github.com/sanger-tol . Table 5 lists software versions used in this study.
