# Supplementary material for: The genome sequence of the Orange Ripple Bryozoan, Schizoporella japonica Ortmann, 1890 (Cheilostomatida: Schizoporellidae)
Source: Wellcome Open Res. 2026 Aug 27;11:556. [Version 1] doi: 10.12688/wellcomeopenres.27394.1 (PMC13646541; doi:10.12688/wellcomeopenres.27394.1)
Supplement: Peer review history

# Reviewer report: Reviewer response for version 1

**DOI:** 10.21956/wellcomeopenres.30149.r168838

**Published:** 2026-10-07

**Authors:** Manuel Curto

**Affiliations:** 1 Boku University, Vienna, Austria

The genome note provides a comprehensive description of the sequencing and assembly of the bryozoan *Schizoporella japonic*a genome. The methodology is appropriate, and the resulting assembly appears to be of relatively high quality.

 I have two suggestions for improvement:

1. **Specimen identification:** Please provide more detail on how the specimen was identified. Was morphological identification performed before molecular confirmation? Are there closely related species in the sampling area that could make identification ambiguous, even with DNA barcoding?
2. **Genome annotation:** Was gene annotation performed? Given that RNA-seq data were generated, these could support gene prediction and annotation. If annotation was not undertaken, please clarify whether it is planned.

Are sufficient details of methods and materials provided to allow replication by others?

Yes

Is the rationale for creating the dataset(s) clearly described?

Yes

Are the datasets clearly presented in a useable and accessible format?

Yes

Are the protocols appropriate and is the work technically sound?

Yes

Reviewer Expertise:

Molecular ecology, population genetics, conservation genetics

I confirm that I have read this submission and believe that I have an appropriate level of expertise to confirm that it is of an acceptable scientific standard.

# Reviewer report: Reviewer response for version 1

**DOI:** 10.21956/wellcomeopenres.30149.r166069

**Published:** 2026-09-16

**Authors:** Hanna Hartikainen

**Affiliations:** 1 University of Nottingham, Nottingham, England, UK

The rationale for the dataset is clear. The species is a widespread marine invasive species, and having a bryozoan genome certainly helps to plug an important data gap. The protocols are appropriate throughout and the assembly metrics are generally very good. Most of the assembly is assigned to chromosomal pseudomolecules, and overall the work appears technically sound.

 For replication purposes, I would be interested in a few more details about sample collection and preparation. It would be useful to mention how the sample was treated to remove associated organisms. Was the colony starved prior to extraction? Were whole-organism samples, including gut contents, used for the DNA extractions? Given the modular nature of bryozoans, it would also be helpful to know roughly how many zooids contributed to each preparation. Finally, were ovicells containing larvae included or excluded from the extracted material?

 One minor point concerns the BUSCO score, which appears a little lower than might be expected for a chromosome-level assembly. This may simply reflect the limited representation of bryozoans in current reference datasets, but a brief comment from the authors would be helpful.

 Overall, this is a very good genome note and a valuable resource. The additional details requested above would improve reproducibility and may also be useful for informing sample collection and preparation for future bryozoan genome projects.

Are sufficient details of methods and materials provided to allow replication by others?

Partly

Is the rationale for creating the dataset(s) clearly described?

Yes

Are the datasets clearly presented in a useable and accessible format?

Yes

Are the protocols appropriate and is the work technically sound?

Yes

Reviewer Expertise:

bryozoa, phylogenetics, ecology

I confirm that I have read this submission and believe that I have an appropriate level of expertise to confirm that it is of an acceptable scientific standard.
